# Isochlorogenic Acid C Alleviates High-Fat Diet-Induced Hyperlipemia by Promoting Cholesterol Reverse Transport

**DOI:** 10.3389/fphar.2022.881078

**Published:** 2022-07-25

**Authors:** Liuyi Zheng, Guangyao Lin, Ruyue Li, Haining Gan, Xuejun Huang, Nan Yao, Dake Cai, Ziming Zhao, Zixuan Hu, Minyi Li, Huazhen Xu, Leyi Li, Sha Peng, Xinxin Zhao, Yijing Lai, Yuxing Chen, Dane Huang

**Affiliations:** ^1^ The Fifth Clinical College of Guangzhou University of Chinese Medicine, Guangzhou, China; ^2^ Guangdong Provincial Key Laboratory of Research and Development in Traditional Chinese Medicine, Guangdong Provincial Second Hospital of Traditional Chinese Medicine (Guangdong Provincial Engineering Technology Research Institute of Traditional Chinese Medicine), Guangzhou, China; ^3^ School of Marxism, Guangzhou University of Chinese Medicine, Guangzhou, China; ^4^ Department of Pharmacy, Zhengzhou People’s Hospital, Zhengzhou, China

**Keywords:** isochlorogenic acid C, hyperlipemia, reverse cholesterol transport, foam cells, cholesterol efflux, inflammatory

## Abstract

**Background:** Promoting cholesterol reverse transport (RCT) has been proven to be a promising hyperlipidemia therapy since it is more effective for the treatment of atherosclerosis (AS) caused by hyperlipidemia. Liver X receptor (LXR) agonists can accelerate RCT, but most of them trigger undesirable liver steatosis due to the activation of liver LXRα.

**Aim:** We aim to figure out whether isochlorogenic acid C (ICAC) facilitates RCT without causing hepatic steatosis.

**Methods:** *In vitro* study, we established foam macrophages and macrophages with loaded NBD-cholesterol models to investigate the competence of RCT promoting ICAC. RT-qPCR and Western blot were used to verify ICAC’s regulation of RCT and NF-κB inflammatory pathways. In this *in vivo* study, male 6-week-old C57BL/6 mice were fed a high-fat diet (HFD) to investigate ICAC’s anti-hyperlipidemic effect and its functions in regulating RCT. The anti-hyperlipidemic effect of ICAC was evaluated by blood and liver lipid levels, liver hematoxylin, oil red o staining, and liver coefficient. Finally, mRNA levels of genes involved in RCT and inflammation pathways in the liver and intestine were detected by RT-qPCR.

**Results:** ICAC prevented macrophages from foaming by up-regulating the LXRα mediated RCT pathway and down-regulating expression of the cholesterol absorption genes LDLR and CD36, as well as suppressing iNOS, COX2, and IL-1β inflammatory factors. In HFD-fed mice, ICAC significantly lowered the lipid level both in the serum and the liver. Mechanistic studies showed that ICAC strengthened the RCT pathway in the liver and intestine but didn’t affect liver LXRα. Furthermore, ICAC impeded both adipogenesis and the inflammatory response in the liver.

**Conclusion:** ICAC accelerated RCT without affecting liver LXRα, thus resulting in a lipid-lowering effect without increasing liver adipogenesis. Our results indicated that ICAC could be a new RCT promoter for hyperlipidemia treatment without causing liver steatosis.

## Introduction

Hyperlipidemia is a chronic lipid metabolism disorder characterized by abnormally elevated serum lipid levels, such as total cholesterol, LDL cholesterol, and triglycerides (Geisler and Renquist, 2017). In the United States, more than 100 million people (about 53% of adults) have elevated LDL-C levels, putting them at a significantly increased risk of developing atherosclerotic cardiovascular disease (ASCVD) (Shimizu et al., 2019). Reducing cholesterol level has been proved to be significantly reduce myocardial infarction, ischemic stroke events and cardiovascular death, improve the quality of life of patients with cardiovascular disease, and effectively reduce the burden of disease (Shimizu et al., 2019). Currently, lipid-lowering drugs are used in clinical trials including 3-hydroxy-3-methylglutaryl-CoA reductase inhibitors (Statins), peroxisome proliferator-activated receptor alpha agonists (fibric acid derivatives), bile acid chelators (niacin), and cholesterol absorption inhibitors (ezetimibe) (Cote et al., 2019). However, even the most effective statins do not achieve a satisfactory endpoint clinical benefit due to their low peripheral cholesterol efflux ability (Kini et al., 2017). Statins markedly reduce acute cardiovascular events (Rea et al., 2018), but only modestly reduce stenosis (Duivenvoorden and Fayad, 2011).

Reverse cholesterol transport (RCT) is a key process of removing excess cholesterol from peripheral tissues, especially cholesterol in macrophages by high-density lipoprotein (HDL) or non-HDL, to the liver and small intestine for excretion (Fisher et al., 2012; Pownall et al., 2021). Our previous study has shown that the RCT pathway is inhibited in hyperlipidemia (Zeng et al., 2016). Ineffective RCT will lead to an increase in circulating cholesterol, followed by a large number of foam cells and aggravate organism microenvironment inflammation (Zhang et al., 2021).

Increasing evidence has demonstrated that promoting RCT is believed to be an important strategy to treat hyperlipidemia (Hirata et al., 2017; Wang et al., 2017). However, there is no agent that targets this mechanism that has been successfully developed for clinical use until now. The liver X receptor (LXR) plays a critical role in the RCT pathway (Thomas et al., 2018; Li et al., 2019). Targeting activation of LXR significantly enhances RCT-related gene expression, including ATP-binding cassette transporter (ABC) A1, ABCG1, ABCG5, ABCG8, scavenger receptor B type 1 (SR-BI) and cholesterol 7α-hydroxylase (CYP7A1) (Pizzini et al., 2017; Peng et al., 2019). Moreover, LXR activation can also have an inhibitory effect on the inflammatory response (Thomas et al., 2018). Therefore, over the past 20 years, developing LXR agonists has been used as the most effective strategy for RCT promoting. Nevertheless, most synthetic LXR agonists induce lipogenic genes like sterol regulatory element-binding protein (SREBP-1c), acetyl CoA carboxylase (ACC), and steroyl CoA desaturase 1 (SCD1) in the liver, causing steatosis (Hong and Tontonoz, 2014; Kirchgessner et al., 2016). Hence, it is necessary to find a substitution that regulates the RCT pathway without causing liver steatosis and hypertriglyceridemia to prevent AS induced by hyperlipidemia.

Isochlorogenic acid C (ICAC), an active compound comes from Yinlantiaozhi capsule which has been shown to have effects in treating hyperlipidemia (Zhuo et al., 2018). Previous research revealed Yinlantiaozhi inhibits macrophage foaming through enhancing LXRα-ABCA1 pathway and suppressing of inflammatory response (Huang et al., 2018). In previous study, we also predicted the pharmacodynamic substance and target of Yinbluanzhiqi capsule by using a computer-aided drug design method. The results found that quinic acid compounds, including ICAC, have a potential role in regulating RCT (Liuyi Zheng et al., 2022). ICAC is a main component of propolis, which has the good effect of lowering blood lipids and suppressing inflammation (Ji et al., 2021). These findings suggest that the ICAC may be an RCT promotor. This study aimed to evaluate the RCT promoting effectiveness of ICAC and to validate the hypolipidemic effect *in vivo*. To achieve this, we first used cholesterol efflux promotion and foam cell inhibition to investigate the RCT activity of ICAC in macrophages. Further, we tested whether ICAC could regulate lipid metabolism by regulating the RCT pathway and ameliorate steatosis in HFD-fed mice.

## Material and Methods

### Bone Marrow-Derived Macrophage Isolation and Induction

Tibial bone marrow cells from 3-month-old C57BL/6 mice (Guangdong Medical Laboratory Animal Center, China) were flushed and suspended in MEM-α (Gibco, UAS). Cells were collected by centrifugating at 500 RCF for 5 min and then cultured in MEM-αcontaining 10% FBS (Gibco, UAS), 1% penicillin–streptomycin (Corning, United States) and 30 μg/ml macrophage-colony stimulating factor (M-CSF) (Proteintech, United States) in a CO_2_ incubator overnight. The adherents were bone marrow-derived macrophages (BMMs), which were digested into cell suspensions by trypsin after successful culture and used in subsequent experiments.

### Cell viability assay

BMMs cells were seeded in a 96-well culture plate with 5 × 10^3^ cells/well and cultured for 24 h. Then, BMMs in 96-well culture plates were treated with 50, 25, 12.5, 6.25, 3.13, and 1.56 μM of ICAC for 24 h. The cell viability was determined by the MTT assay as we previously described (Huang et al., 2020).

### Ox-LDL Induced Macrophage Foam Cells and Detection

RAW264.7 5 × 10^3^ cells/well were diluted with complete medium and seeded on a 96-well culture plate for 24 h. After that, cells were treated with ox-LDL at a final concentration of 50 μg/ml to generate foam cells. In the meantime, the cells were indicated with or without compounds for 24 h. 0.1% DMSO was used as a control in the group that was without compound treatment. Foam cells were detected by oil red O staining (ORO) according to the reported method. Briefly, the supernatant was removed and the cells were fixed with 4% paraformaldehyde and stored at 4°C overnight. The cells were washed three times with PBS after the paraformaldehyde was discarded and stained with an oil red O staining kit (Nanjing Jiancheng Bioengineering Research Institute, China). The foam cell was observed under a microscope (Leica DMi8/DPC7000T, Germany). Intracellular lipids were extracted by adding 50 μl of isopropyl alcohol and then shaking for 10 min. The extracted lipids were detected at a 492 nm wavelength by an automatic microplate reader (Thermo Varioskan Flash, United States).

For RT-qPCR and analyses, BMMs cells were diluted with complete medium containing 10 μg/ml M-CSF and seeded on a 6-well culture plate. BMMs were treated with 10 μM ICAC for 24 h, after stimulated by ox-LDL (50 μg/ml) for 24 h. Then all the cells were washed twice by PBS and collected for RT-qPCR or Western bolt experiments.

### Cholesterol Efflux Assay

RAW264.7 cells were plated according to the method mentioned above. After 24 h of culture, RAW264.7 was equilibrated with 2 μM 25 NBD-cholesterol for 24 h and the lipid overload model of RAW264.7 was obtained. Subsequently, the cells were washed with PBS twice, and then cultured with serum-free culture with ICAC at 25, 12.5, 6.25, 3.13, and 1.56 μM for 18 h. HDL (final concentration of 30 μg/ml) was added, while the control wells were left without HDL for 2 h. The fluorescence-labeled cholesterol was released from cells into the medium and then was measured using a multifunctional enzyme plate apparatus at 485 nm excitation and 535 nm emission light by an automatic microplate reader. The fluorescence cholesterol content in the cell supernatant of treatment minus the cholesterol in the supernatant of control well was taken as the index to measure the cholesterol efflux of cells. The higher the ratio was, the higher the external cholesterol efflux was.

### *In Vivo* Study

All animal experiments were approved by the Animal Ethics Committee of the Guangdong Provincial Engineering Technology Institute of Traditional Chinese Medicine (Guangzhou, China). The 6-week-old male C57BL/6 mice weighing 18∼22 g were obtained from the Guangdong Medical Experimental Animal Center (Guangzhou, China). C57BL/6 mice were randomly separated into control group, high-fat diet (HFD) (SYSE Bio-tec Co., Ltd., China) group, HFD with 40 mg/kg/d atorvastatin group (AT; positive control drug), HFD with 2 mg/kg/d T0901317 (T090, APExBIO, United States) group, HFD with 20 mg/kg/d ICAC (Chengdu, China) group, and HFD with 10 mg/kg/d ICAC combined with 2 mg/kg/d T0901317 group. Except for the control group, the others were fed a high-fat diet supplemented with the corresponding drug for 12 weeks. Mice were sacrificed at the end of the treatment. The liver, brown adipose tissue, kidney, heart, and body weight were recorded. The serum was collected to detect the content of total cholesterol (TC), high-density lipoprotein (HDL-C), low-density lipoprotein cholesterol (LDL-C), and ox-LDL. Parts of livers and intestines were stored at −80°C for mRNA and lipid detection. Livers for hematoxylin-eosin (HE) staining and ORO staining were fixed in paraformaldehyde.

### Organ Coefficient Determination

At the end of the treatment, the body weight was recorded. And all the organs were removed from the attached adipose and weighted with an electronic balance (Sartorius, Germany). The organ index (mg/g) was calculated as the following formula:

$$\mathrm{Organ}\;\mathrm{coefficients}=\frac{Organ\;weight\left(mg\right)}{Rat\;or\;mouse\;total\;weight\left(g\right)}.$$

### Lipid Detection

Liver TC and TG were extracted by using the organic solvent extraction method. 100 mg of liver were grinded with 1 ml of physiological saline. The homogenate was divided into two parts, one was used to detect BCA, and the other was added to 500 μl of organic solvent (methanol: chloroform = 2:3), 12,000 g, 10 min, to remove the clear. Volatilize the organic solvent overnight. TC and TG can both be detected by using commercial assay kits (Nanjing Jiancheng Bioengineering Research Institute, China). Serum TC, HDL-C, and LDL-C levels were measured by commercial assay kits (Nanjing Jiancheng Bioengineering Research Institute, China), ox-LDL levels were measured by ELISA assay according to the manufacturer’s protocol (Tianjin Anoric Bio-technology Co., Ltd., China).

### Liver Hematoxylin-Eosin and Oil Red O Staining

Livers were fixed in 4% paraformaldehyde (Servicebio, China) and made into slices (Thermo) for HE staining and ORO staining. For HE, the paraffin sections were dewaxed to water, stained with hematoxylin (Servicebio, China) and eosin (Servicebio, China), then dehydrated and sealed. For ORO, reheat and dry the frozen slices, then fix them in the fixative solution for 15 min, wash with tap water, and dry. Stain sections with Oil Red solution for 8–10 min in the dark, and cover them with a lid during dyeing (Servicebio, China). Differentiation of background was followed by immersion in hematoxylin. Seal the slices with glycerin gelatin. HE and ORO staining were observed under the microscope.

### RT–qPCR Analysis

Total RNA isolation and mRNA levels for specific genes in macrophages, livers and intestine were performed using a real-time quantitative polymerase chain reaction (RT-qPCR) assay. Total RNA was extracted using Trizol reagent (Thermo Fisher Scientific, United States). cDNA was synthesized with 0.25 μg total RNA by using the Evo M-MLV RT Premix for qPCR (Accurate Biology, China). DNA amplification was performed using the SYBR Green Premix Pro Taq HS qPCR Kit (Accurate Biology, China) with 0.5 μl of ROX reference dye (Accurate Biology, China) and performed in a StepOnePlus Real-Time PCR system (Thermo Fisher Scientific, United States) using SYBR Green detection chemistry with the resulting cDNAs. The primers sequences used were shown in Table 1.

**TABLE 1:** Amplified gene primers.

Primer	Sense (5′ - 3′)	Antisense (5′ -3′)	Genebank	bp
LXR	CAA​TGC​CTG​ATG​TTT​CTC​CTG​A	GCT​GAC​TCC​AAC​CCT​ATC​CCT​A	NM_013839.4	154
ABCG1	GCC​TAC​TAC​CTG​GCA​AAG​AC	GAG​CAG​CGA​ACA​GCA​CAA​AA	NM_009593.2	130
ABCA1	AGC​AAG​ACG​AAA​CAG​ACG​GG	GGG​CAA​TGC​AAA​CAA​AGA​CA	NM_013454.3	284
SR-BI	CTG​GTG​GAC​AAA​TGG​AAC​GG	TGG​CAA​ACA​GAG​TAT​CGG​GG	NM_016741.2	232
CD36	AGC​CTG​TGT​ATT​ATT​TCG​CT	TAT​GTT​GAC​CTG​CAG​TCG​TT	NM_001159558.1	165
LDLR	GAC​TGG​TCA​GAT​GAA​CCC​ATC​AAA​G	AGG​TCA​TTG​CAG​ACG​TGG​GAA​C	NM_010700.3	86
ABCG5	TGG​ATC​CAA​CAC​CTC​TAT​GCT​AAA	GGC​AGG​TTT​TCT​CGA​TGA​ACT​G	NM_031884.2	77
ABCG8	TGC​CCA​CCT​TCC​ACA​TGT​C	ATG​AAG​CCG​GCA​GTA​AGG​TAG​A	NM_026180.3	73
SCD-1	TTC​TTG​CGA​TAC​ACT​CTG​GTG​C	CGG​GAT​TGA​ATG​TTC​TTG​TCG​T	NM_009127.4	98
ACC	AGG​ATT​TGC​TGT​TTC​TCA​GAG​CTT	CAG​GAT​CTA​CCC​AGG​CCA​CAT	NM_133360.3	141
FAS	GGC​TCT​ATG​GAT​TAC​CCA​AGC	CCA​GTG​TTC​GTT​CCT​CGG​A	NM_007988.3	190
SREBP-1c	CAG​CTC​AGA​GCC​GTG​GTG​A	TGT​GTG​CAC​TTC​GTA​GGG​TC	NM_011480.4	225
CYP7A1	TCA​AGA​CCG​CAC​ATA​AAG​CC	GAG​ATG​CCC​AGA​GGA​TCA​CG	NM_007824.3	177
NF-κB	GCT​CCC​GAA​ACC​AAT​CTT​AC	AGC​TCC​CAA​TTC​TCC​AAT​CC	NM_025937.4	230
IL-1β	GCA​ACT​GTT​CCT​GAA​CTC​AAC​T	ATC​TTT​TGG​GGT​CCG​TCA​ACT	NM_008361.4	89
iNOS	GCAGCTGGGCTGTACAAA	AGC​GTT​TCG​GGA​TCT​GAA​T	NM_010927.4	84
COX2	ATG​CTC​CTG​CTT​GAG​TAT​GT	CAC​TAC​ATC​CTG​ACC​CAC​TT	NM_011198.4	696
β-actin	GCA​ACT​GTT​CCT​GAA​CTC​AAC​T	ATC​TTT​TGG​GGT​CCG​TCA​ACT	NM_008361.4	89

### Western Blot Analysis

For the WB experiment, cells were lysed in RIPA Lysis buffer containing PMSF (Beibokit, China). SDS-polyacrylamide gel electrophoresis was used to separate equal amounts of proteins from whole-cell lysates and then transferred onto PVDF membranes (Bio-Rad, United States). The blots were probed with various primary antibodies: LXRα (Santa Cruz, United States), ABCA1 (Abcam, United Kingdom), ABCG1 (Abcam, United Kingdom), Cyclooxygenase 2 (COX2) (Abcam, United Kingdom), Interleukin 1 beta (IL-1β), SR-BI, inducible nitric oxide synthase (iNOS) (Abcam, United Kingdom), fatty acid translocase (also known as cluster of differentiation 36, CD36) (Affinity, Australia), and low density lipoprotein receptor (LDLR) (Affinity, Australia), after being blocked with 8% (w/v) skimmed milk and then the appropriate secondary antibodies (1:10,000). After washing the membrane, it is developed by chemiluminescence in the ECL reagents (Thermo Fisher Scientific, United States), and the gel imaging analysis system (Tanon 5220 multi, China) was used for camera analysis. The target band of the obtained protein image and the gray value of the corresponding internal reference band were measured by Image J software.

### Statistical analysis

All experiments were performed independently at least three times. The results are shown as the mean ± SEM. A one-way ANOVA test was used to compare differences between groups in the various experiments. Differences with a *p*-value < 0.05 were considered statistically significant.

## Results

### Isochlorogenic Acid C Triggers Reverse Cholesterol Transport Pathway and Promotes Cholesterol Efflux

**FIGURE 1:**
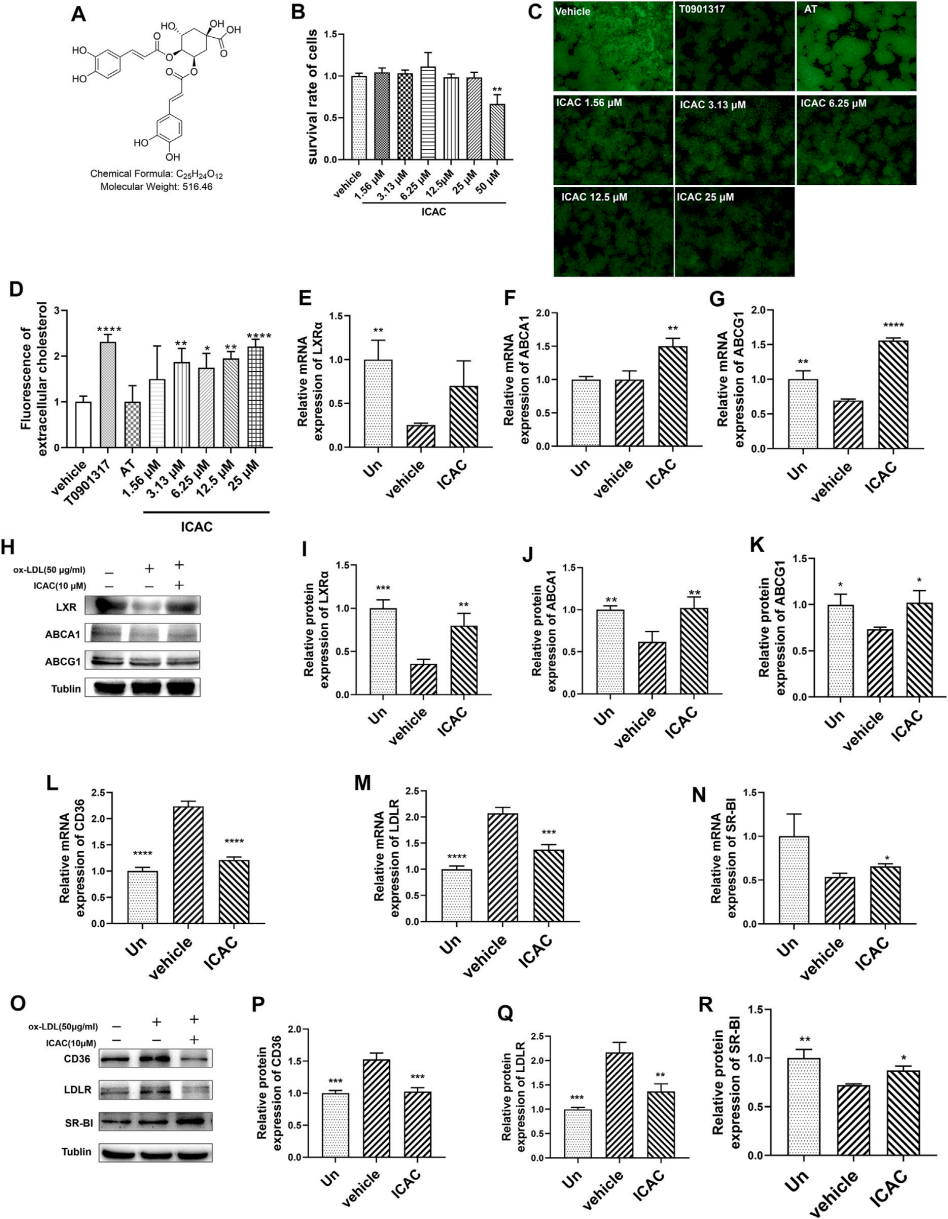
ICAC triggers the reverse cholesterol transport pathway and promotes cholesterol efflux. **(A)** Chemical structure of isochlorogenic acid C (ICAC). **(B)** Cell survival rate assay of ICAC in BMMs. **(C)** Fluorescence in BMMs under light microscopy. **(D)** Extracellular fluorescent cholesterol intensity was measured with a microplate reader. **(E–G)** Gene expression level of LXRα, ABCA1 and ABCG1 in BMMs. **(H–K)** Protein expression level of LXRα, ABCA1, and ABCG1 in BMMs. **(L–N)** Gene expression level of CD36, LDLR, and SR-BI in BMMs. **(O–R)** Protein expression level of CD36, LDLR, and SR-BI in macrophages. All data in **(B,D–G,I–N,P–R)** were represented as mean ± SEM. Statistical difference was determined by one -way ANOVA test. Compare with a vehicle, ^*^ *p* < 0.05, ^**^ *p* < 0.01, ^***^ *p* < 0.001, ^****^ *p* < 0.0001. *n* = 3 for each group.

Foam cells are characterized by cholesterol overload. Excessive cholesterol in macrophages needs to be excreted from cells by the RCT process to avoid foam cells forming. In this study, NBD-cholesterol was applied to prepare cholesterol-overloaded macrophages (Sengupta et al., 2013). To figure out the potential contribution of ICAC (Figure 1A) to cholesterol efflux, we initially evaluated the cytotoxicity of ICAC on BMMs. The results suggested that ICAC had no significant cytotoxicity at a concentration lower than 50 μM (Figure 1B). Cholesterol uptake results show that macrophages uptake NBD-cholesterol. The NBD-cholesterol load-macrophages are presented by green fluorescence. (Figure 1C). In order to detect the cholesterol efflux ability of ICAC, NBD-cholesterol load-macrophages were treated with different concentrations of ICAC. ICAC significantly promoted cholesterol efflux in a dose-dependent manner. As shown in Figures 1C,D, NBD-cholesterol fluorescence in macrophages was significantly reduced when ICAC≧ 3.13 μM. In addition, the results also show that LXR agonist T0901317 also increased cholesterol efflux, while HMG-COA inhibitor AT had no significant effect (Figures 1C,D).

Due to the major regulatory functions of LXRs and their downstream genes on lipid homeostasis, especially cholesterol metabolism and efflux, we aimed to evaluate whether ICAC affected the LXR-mediated RCT signaling pathway. According to the results of RT-qPCR and Western blot assays, ICAC increased the expression of genes and proteins of the RCT signaling pathway, including LXRα, ABCA1 and ABCG1 in macrophages (Figures 1E–K). ABCA1 and ABCG1 are upregulated by the activation of LXR, which promotes the efflux of free cholesterol from foam cells (Guo et al., 2018). Hence, a reduction of intracellular NBD-cholesterol fluorescence was observed after ICAC treatment (Figures 1C,D). Meanwhile, ICAC suppressed the cholesterol uptake association genes CD36 and LDLR and increased SR-BI (Figures 1L–R). These data indicate that ICAC inhibits cholesterol absorption and enhances the RCT pathway.

### Isochlorogenic Acid C Inhibits Oxidized Low-Density Lipoproteins-Induced Foam Macrophage Formation and Inflammatory Response

**FIGURE 2:**
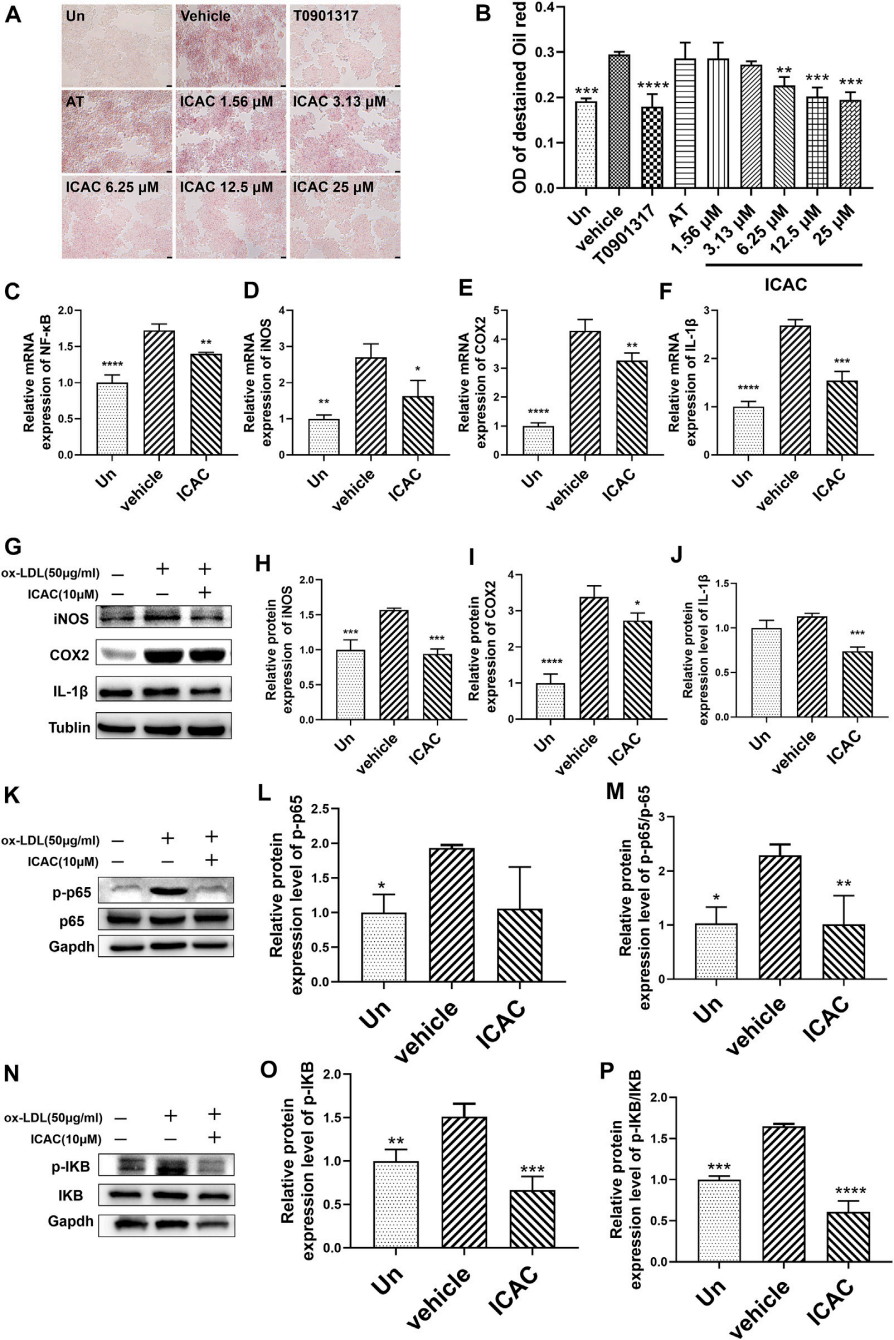
ICAC inhibits ox-LDL-induced foam macrophage formation and inflammatory response. **(A)** Oil red O staining of lipid accumulation in BMMs stimulated by ox-LDL. **(B)** Quantification of ORO staining in BMMs (*n* = 3). **(C–F)** Gene expression level of NF-κB, iNOS, COX, and IL-1β in BMMs. **(G–J)** Protein expression level of iNOS, COX2, and IL-1β in BMMs. **(K–M)** Protein expression level of NF-κB pathway. All data in **(B–F, H–J, N, M, O, P)** were represented as mean ± SEM. Statistical difference was determined by one -way ANOVA test. Compare with a vehicle, ^*^ *p* < 0.05, ^**^ *p* < 0.01, ^***^ *p* < 0.001, ^****^ *p* < 0.0001. *n* = 3 for each group.

Ox-LDL is characterized as a harmful type of cholesterol which leads to the formation of foam cells. Enhancing the cholesterol efflux capacity in macrophages is considered to play an important role in inhibiting the formation of foam cells as well as reducing the inflammatory response (Mitchell and Carmody, 2018). Hence, we first investigated the competence of macrophages’ foaming inhibition of ICAC at 25 μM, 12.5 μM, 6.25 μM, 3.13 μM , and 1.56 μM in ox-LDL-stimulated RAW264.7. ORO staining results showed that ICAC and T0901317 significantly prevented cell foaming and intracellular lipid accumulation (Figures 2A,B).

NF-κB is a crucial transcription factor in directing the initiation and progression of inflammation. It controls expression of genes including cytokines IL-1β, iNOS, COX2 (Mitchell and Carmody, 2018). BMMs obtained from C57BL/6 mice were used to induce inflammation in macrophages. Next, inflammatory pathway related genes and proteins were evaluated. After treating with ICAC in ox-LDL-induced inflammatory macrophages, the expression mRNA level of NF-κB (Figure 2C). Meanwhile, the mRNA and protein levels of NF-κB downstream inflammatory factors iNOS, COX2 and IL-1β were decreased significantly (Figures 2D–J). Further studies have shown that ICAC inhibits the phosphorylation of P65 (Figures 2K–M) and IKB (Figures 2N–P), which indicates that the suppressing of the NF-κB signaling pathway. These results suggest that ICAC plays a role in inhibiting ox-LDL-induced foam cell formation and NF-κB pathway related inflammation responses.

### Isochlorogenic Acid C Decreases Serum Cholesterol and Increases Bile Acid Levels in Hyperlipidemia Mice

**FIGURE 3:**
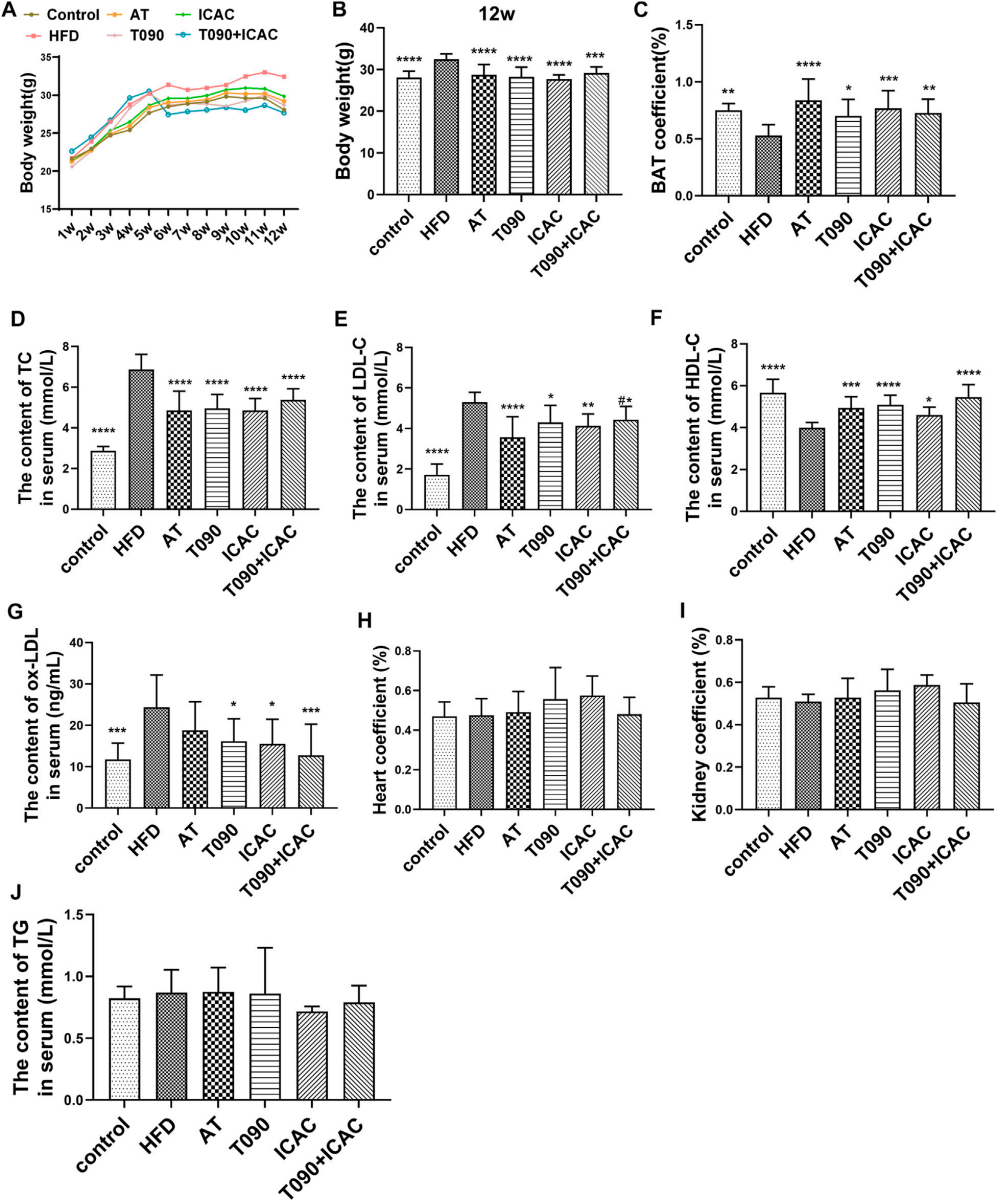
ICAC decreases serum cholesterol and increases bile acid levels in hyperlipidemic mice. **(A)** Weekly weight of mice in each group. **(B)**Weight of each group in the 12-week. **(C)** Coefficients of brown adipose tissue. **(D–G)** Serum TC, LDL-C, HDL-C, ox-LDL of C57BL/6 mice fed a chow diet or a high fat diet with or without T0901317 or icac for 12 weeks. **(H,I)** Coefficients of the heart and kidney. **(J)** Serum TG levels of all groups at the end of treatment. All data were represented mean ± SEM. Statistical difference was determined by one -way ANOVA test. Compare with HFD, ^*^ *p* < 0.05, ^**^ *p* < 0.01, ^***^ *p* < 0.001, ^****^ *p* < 0.0001. *n* = 10 for each group. Compared with AT, ^#^ *p* < 0.05.

Next, the *in vivo* lipid-lowering effect of ICAC was evaluated by using HFD-induced hyperlipidemia in C57BL/6 mouse. Two positive drugs were carried out in this study. One is the cholesterol synthesis inhibitor atorvastatin (AT), and the other is the LXR agonist T090. Compared with the rodent chow diet group (control group), the body weight of mice in the HFD group was significantly increased. HFD mice lost their body weights significantly with the treatment of ICAC, T090, AT, and T090 + ICAC (Figures 3A,B). Besides, the brown adipose tissue (BAT) coefficients were increased (Figure 3C), which may explain the HFD mice weight reduction after treatment with ICAC, T090, AT, and T090 + ICAC. Additionally, ICAC reduced serum TC (Figure 3D) and LDL-C (Figure 3E) in hyperlipidemia mice, as well as improved serum HDL-C level as two positive drugs did (Figure 3F). The data also indicated that ICAC can reduce the content of ox-LDL (Figure 3G), a high-risk factor for atherosclerosis. Our research also found that a half dose of ICAC combined with T090 own equal therapeutic effective compared to a single high dose of themselves respectively (Figures 3A–G). Furthermore, groups of ICAC, T090 + ICAC, and T090 but not AT showed significantly ox-LDL lowering effects (Figure 3G). Meanwhile, kidney coefficient and heart coefficient had no significant change, that mean ICAC had no toxicity to these organs at 20 mg/kg dose (Figures 3H,I). In serum pharmacodynamics parameters, ICAC has no significant difference in therapeutic effect from AT and T090 (Figure 3). These results suggest that ICAC is a potential drug for RCT promoting that can be used to treat hyperlipemia.

### Isochlorogenic Acid C Decreases Hepatic Tissues Lipid and Inflammation Levels in High-Fat Diet-Induced Hyperlipidemia Mice

**FIGURE 4:**
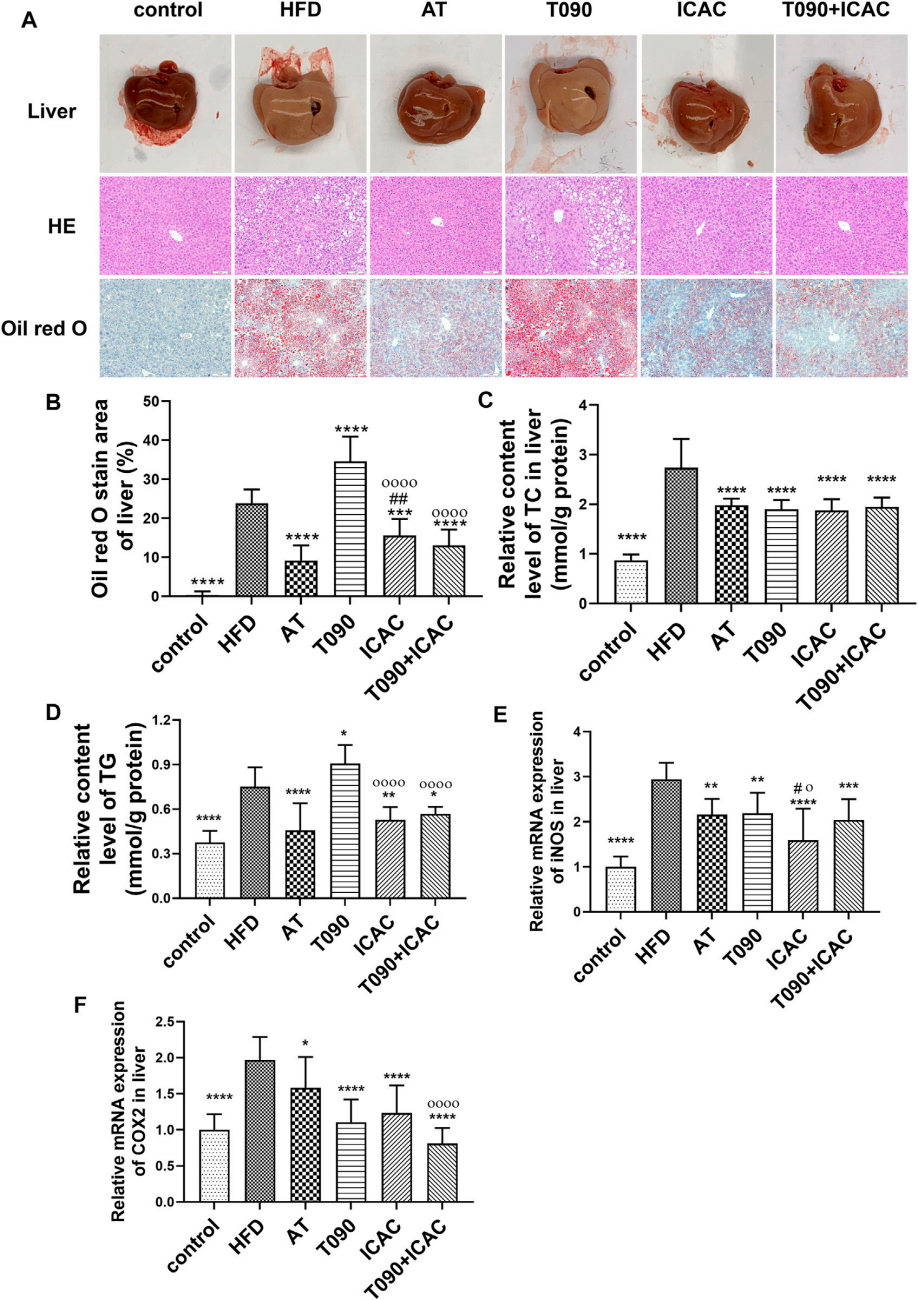
ICAC decreases hepatic tissue lipid and inflammation levels in HFD-induced hyperlipidemic mice. **(A)** Liver morphological investigation, HE and ORO staining of liver biopsy. **(B–F)** ORO staining area, TC, TG level and the gene expression levels of iNOS and COX2 in liver of C57BL/6 mice fed a chow diet or a high fat diet with or without AT, T0901317 or ICAC for 12 weeks. All data in **(B–F)** were represented mean ± SEM. Statistical difference was determined by one -way ANOVA test. Compared with HFD, ^*^ *p* < 0.05, ^**^ *p* < 0.01, ^***^ *p* < 0.001, ^****^ *p* < 0.0001. Compared with AT, ^#^ *p* < 0.05, ^##^ *p* < 0.01. Compared with T090, ^ο^ *p* < 0.05, ^οοοο^ *p* < 0.0001. *n* = 10 for each group.

To further investigate the therapeutic effect of ICAC on HFD-induced hyperlipidemia in mice, liver morphological observations and lipid and inflammatory factor detection were performed. In the HFD group, the livers were light brown and the edges were passive and greasy (Figure 4A). HE staining showed that liver cells in the HFD group were swollen, cytoplasm was loose, lipid droplets of different sizes were vacuolated, and cell boundaries were unclear (Figure 4A). The ORO staining assay further showed increased lipid staining area of liver cells in the HFD group (Figures 4A,B). Compared with the HFD group, the liver color of treatment groups (ICAC, AT, and ICAC + T090) was redder, with sharp edges and smooth surfaces. In addition, the vacuoles and ordered cells were reduced after drug treatment in HE staining (Figure 4A). Furthermore, the number of cells stained by ORO were also significantly reduced after treating with ICAC, AT, and T090 + ICAC (Figures 4A,B). Additionally, TC contents of the liver were all inhibited in all treatment groups (Figure 4C). But unlike AT and ICAC, T090 significantly increased liver vacuolar lesions, ORO-stained lipid area, and TG content (Figures 4A,B,D), which indicated T090 had induced liver steatosis. Interestingly, ICAC enabled to eliminate the undesired liver steatosis of T090 when T090 combination with ICAC was used in treating hyperlipemia mice (Figures 4A,B,D). With the decrease of lipid in the liver, we also found that groups of ICAC, T090, AT, and T090 + ICAC displayed inhibitory effects on the gene expression of iNOS and COX2 in the liver (Figures 4E,F). In this study, the concent of TG in serum in all groups did not Significant change (Figure3J).

### Isochlorogenic Acid C Up-Regulates Liver and Intestine RCT Pathway Without Inducing Lipogenesis Gene Expression

**FIGURE 5:**
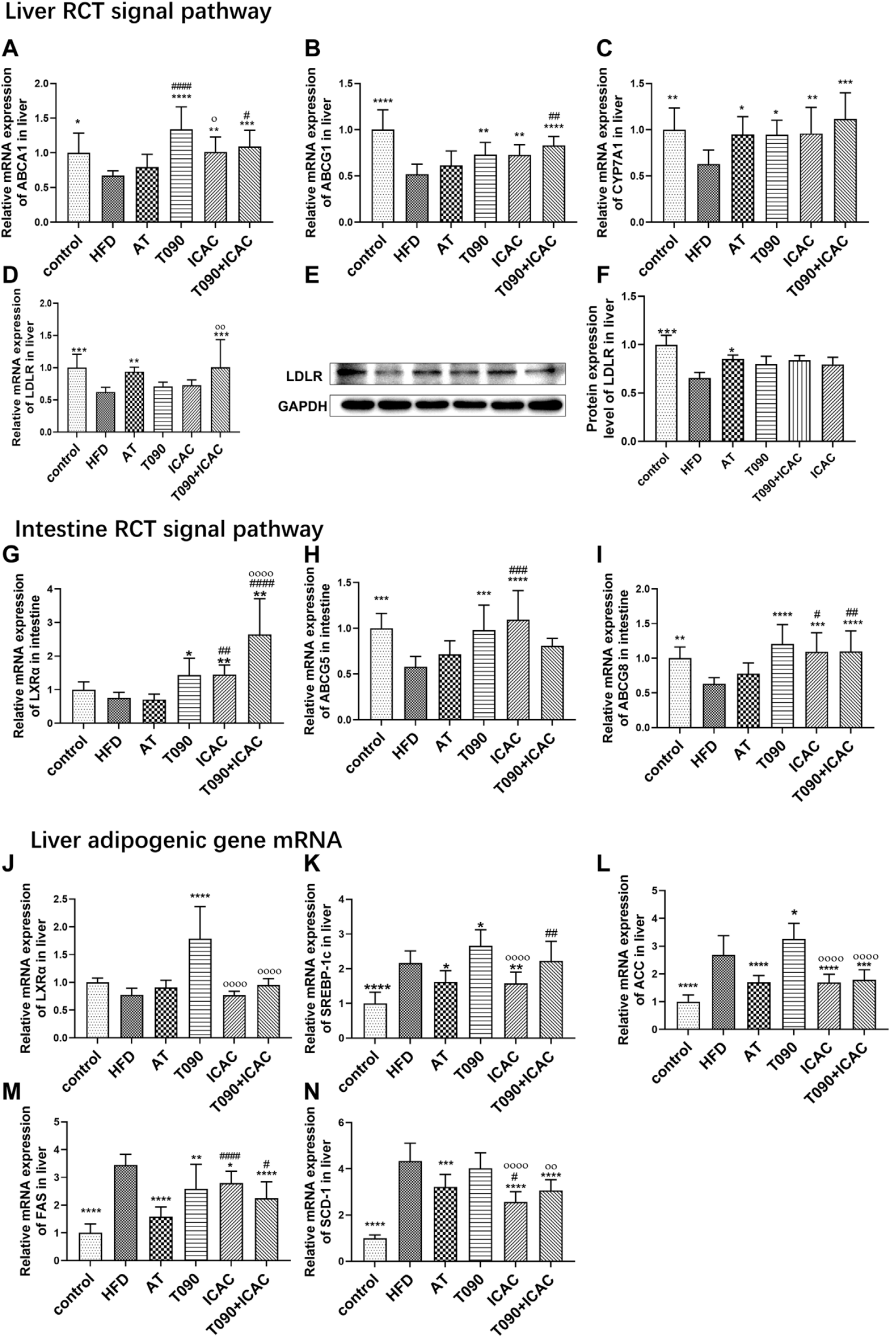
ICAC upregulates liver and intestine RCT pathways without inducing lipogenesis gene expression. **(A–C)** Gene expression levels of ABCA1, ABCG1, and CYP7A1, in the liver of mice. **(D–F)** Gene and protein expression levels of LDLR in the liver of mice. **(G–I)** Gene expressions levels of LXRα, ABCG5, and ABCG8 in the intestine of mice. **(J–N)** Gene expression levels of LXRα, SREBP-1c, ACC, FAS, and SCD-1 in the liver of mice. All data were represented mean ± SEM. Statistical difference was determined by one -way ANOVA test. Compared with HFD, ^*^ *p* < 0.05, ^**^ *p* < 0.01, ^***^ *p* < 0.001, ^****^ *p* < 0.0001. Compared with AT, ^#^ *p* < 0.05, ^##^ *p* < 0.01. Compared with T090, ^ο^ *p* < 0.05, ^οοοο^ *p* < 0.0001. *n* = 10 for each group.

Next, we investigated the effects of ICAC in regulating RCT and adipogenesis pathways *in vivo*. ICAC but not AT up-regulated the expression of RCT key genes, including ABCA1, ABCG1, and CYP7A1 in the liver (Figures 5A–C), as well as increased LXRα, ABCG5, and ABCG8 in the intestine (Figures 5G–I). In addition, another key gene in the RCT pathway, LDLR, was also observed to have a trend of up-regulation after treatment (Figures 5D–F). These results indicate that ICAC promotes RCT.

A previous study explained that T090 activates LXRα and follows by amplifying expression of its downstream target gene SREBP-1c, leading to hepatic and plasma triglyceride levels increasing (Kim et al., 2021). In our study, T090 significantly increased LXRα mRNA level (Figure 5J), and caused increasing of lipogenic genes SREBP-1c and ACC in the liver (Figures 5K,L). However, ICAC and AT did not increase the liver LXRα mRNA level (Figure 5J). Moreover, both ICAC and AT inhibited the expression of SREBP-1c (Figure 5K), as well as its downstream genes like FAS, ACC, and SCD-1(Figures 5L–N). In addition, the combination of T090 with ICAC also significantly reduced the expression of adipogenic genes, FAS, ACC, and SCD-1 (Figures 5L–N). In conclusion, ICAC performed multiple functions, such as RCT promoting, lipogenesis suppressing, as well as inflammatory inhibiting. Compared to AT, ICAC had the advantage of facilitating RCT. Compared to T090, ICAC did not cause undesirable liver adipogenesis.

## Discussion

Targeting LXR activation produces potent RCT-promoting and anti-inflammatory effects (Thomas et al., 2018; Li et al., 2019). In the past 20 years, a number of attempts have been made to facilitate RCT through activating LXR, but all progress has been hindered due to LXR agonists causing undesirable liver steatosis and hypertriglyceridemia (Hong and Tontonoz, 2014; Kirchgessner et al., 2016). These side-effects were caused by upregulation of LXRα and its downstream adipogenic genes, including FAS, ACC, SREBP-1c, and SCD-1 (Jiao et al., 2018).

This study revealed that ICAC acts in a role like synthetic LXR agonists, which promotes the whole-body RCT. However, unlike most LXR agonists, ICAC can treat hyperlipidemia without liver adipogenesis increasing because it has no effect on liver LXRα. Our results figured out that ICAC accelerates the whole body RCT in the following ways: 1) ICAC increases LXRα and its downstream genes ABCA1, ABCG1, and SR-B1 in macrophages, which mediate the efflux of cholesterol and phospholipids to high density lipoprotein (HDL) particles (Fruchart et al., 1994; Ohashi et al., 2005). 2) ICAC only upregulates ABCA1, ABCG1, and CYP7A1 genes in the liver RCT pathway, but doesn’t affect the LXRα gene in the liver. Cholesterol is secreted by hepatic transporters ABCA1 and ABCG1 or metabolized into bile acids by CYP7A1 for eventual excretion (Fruchart et al., 1994; Ohashi et al., 2005). 3) In the intestine, ICAC also strengthens the genes expression of LXRα and ABCG5/8, which inhibit the excessive intake of cholesterol and further reduce whole body lipid level (Fruchart et al., 1994; Ohashi et al., 2005; Zhao et al., 2019).

A previous study showed that LXR agonist T090 treatment at 25 mg/kg/d induced steatosis and hypertriglyceridemia (Kim et al., 2021). Therefore, we applied T090 at a lower dose (2 mg/kg/d) in HFD mice to avoid the side-effect. Our results found T090 can cause liver steatosis at a concentration of 2 mg/kg/d and increase the key lipogenic factors including SREBP-1c, FAS, and SCD-1. However, significantly decreasing of liver lipogenic genes FAS, ACC, SREBP-1c, and SCD-1 were observed when treated with ICAC in hyperlipidemia mice. Moreover, ICAC can also eliminate the side-effect caused by T090 when treatment of T090 + ICAC. In addition, ICAC significantly suppressed CD36 and LDLR mRNA levels in ox-LDL stimulated macrophages. These results revealed that ICAC can prevent macrophages from uptaking excessive lipid, which is conducive to preventing foam cell formation (Solanki et al., 2018; Tian et al., 2020).

AT is a selective and competitive inhibitor of HMG-CoA and has been widely used to treat cardiovascular diseases by reducing plasma cholesterol and lipoprotein levels (Weng et al., 2010). But through intravascular ultrasound measurement, it was found that AT has a weak effect on inducing coronary atherosclerosis regression (Ferreira and Marques da Silva, 2017). A reasonable explanation is that AT cannot promote RCT. Our study also confirmed that AT had no effect on RCT pathway key gene levels of LXRα, ABCA1 and ABCG1 *in vivo*. This suggests that ICAC may be a better drug for AS treatment due to its RCT promotion effect.

In the pathological state of hyperlipemia, RCT function is affected, resulting in excess lipids accumulating in macrophages and inducing a subsequent inflammatory responsee. (Sano et al., 2004; Li et al., 2020; Li et al., 2021). Lipid-derived macrophage inflammation will further stimulate the foaming of macrophages and increase the level of systemic inflammation by activating the nuclear factor κB (NF-κB) pathway (Plotkin et al., 2017). In this study, we also observed high expression of macrophage inflammatory factors after ox-LDL stimulation. ICAC treatment showed inhibition of the expression of the NF-κB signal pathway as well as inflammatory factors such as NF-κB, COX2, iNOS, and IL-1β, indicating that the reduced inflammatory response of ICAC may be due to the reduced lipid accumulation.

In summary, ICAC upregulates LXRα in macrophages and intestines but does not affect liver LXRα. This causes RCT pathway simulation without increasing liver lipogenesis. *In vivo* experiments further proved that ICAC has an anti-hyperlipidemia effect without causing an increase in triglycerides. ICAC can also inhibit the expression of membrane proteins such as CD36 and LDLR, thereby reducing the continuous absorption of cholesterol in macrophages. Moreover, ICAC can inhibit inflammation by lowering gene expression of iNOS and COX2 in foam macrophages and livers.

## Conclusion

Our study indicates ICAC has benefits in promoting RCT and inhibiting inflammation without increasing hepatic lipogenesis. Unlike LXR agonists, ICAC does not cause triglyceride increases or liver steatosis. ICAC is proved to upregulate LXRα-mediated RCT pathway in macrophages and intestine, but does not affect liver LXRα. This study also suggests that ICAC may be a candidate for treating hyperlipidaemia. The anti-hyperlipidemia effect of ICAC is mainly caused by the facilitation of RCT and an anti-inflammatory effect. These findings also suggest that promoting RCT at the signaling pathway level has a potential advantage compared to targeting LXR directly. The findings of this study could offer valuable insights for the discovery of RCT promotor without causing undesirable hypertriglyceridemia and liver steatosis by using existing phytomedicine.

## Data Availability

The original contributions presented in the study are included in the article/supplementary materials; further inquiries can be directed to the corresponding authors.

## Glossary

AS: Atherosclerosis
ox-LDL: oxidized low-density lipoproteins
NF-κB: Nuclear factor κB
iNOS: inducible nitric oxide synthase
COX2: Cyclooxygenase 2
IL-1β: Interleukin 1 beta
RCT: reverse cholesterol transport
LXR: liver X receptor
ABCA1: ATP-binding Cassette Transporter A1
ABCG1: ATP-binding Cassette Transporter G1
ABCG5: ATP-binding Cassette Transporter G5
ABCG8: ATP-binding Cassette Transporter G8
SREBP-1c: sterol regulatory element-binding protein-1c
ACC: acetyl-CoA carboxylase
SCD1: stearoyl-CoA desaturase 1
FAS: fatty acid synthetase
CYP7A1: cytochrome P450 7A1
CD36: cluster of differentiation 36
LDLR: low density lipoprotein receptor
SR-BI: Scavenger Receptor Class B Type 1
TG: triglyceride
TC: total cholesterol
HDL-C: high-density lipoprotein
LDL-C: low-density lipoprotein cholesterol
FBS: fetal bovine serum
HE: hematoxylin-eosin
ORO: oil red O staining
BAT: brown adipose tissue
BMMs: bone marrow-derived macrophages
NBD-cholesterol: 25-{N-[(7-nitrobenz-2-oxa-1,3-diazol-4-yl)-methyl]amino}-27-norcholesterol
M-CSF: macrophage-colony stimulating factor
PMSF: phenylmethylsulfonyl fluoride
MEM α: Minimum Essential Medium α
DMSO: Dimethyl sulfoxide
T090: T0901317
AT: atorvastatin
ICAC: Isochlorogenic acid C
HFD: high fat diet.
